# Mortality rates among non-Hispanic Black and White persons in carbapenemase-producing Enterobacterales, Tennessee, 2015–2019

**DOI:** 10.1017/ash.2022.181

**Published:** 2022-05-16

**Authors:** Erika Kirtz, Rany Octaria, Carolyn Stover, Christopher Wilson, Allison Chan

## Abstract

**Background:** Carbapenem-resistant Enterobacterales (CRE) are an urgent public health threat, particularly those that produce carbapenemase (CP-CRE). Certain risk factors associated with CRE acquisition have been well described, such as older age, indwelling devices, prior hospitalizations, and underlying conditions. However, data are limited regarding the association of CRE and health disparities, such as race and ethnicity. Published literature has consistently shown that minority groups, including but not limited to Non-Hispanic Black persons, have higher risks of developing adverse health outcomes. To better understand the impact of race and ethnicity in CP-CRE cases, we compared 1-year mortality rates among Non-Hispanic Blacks and Non-Hispanic Whites. **Methods:** CRE are reportable in Tennessee; isolates must be sent to the State Public Health Laboratory for carbapenemase detection and resistance mechanism testing. We linked 2015–2019 CP-CRE surveillance cases and laboratory data from our statewide surveillance system, the National Disease Surveillance System (NEDDS)-Base System, with the Tennessee Hospital Discharge Data System (HDDS) and vital records databases. Database linkage and data analyses were performed using SAS version 9.4 software. **Results:** Among 615 CP-CRE cases, the mean age was lower among non-Hispanic Blacks (59 years; SD, 16.6) compared to non-Hispanic Whites (mean, 65 years; SD, 15.7). Among 156 non-Hispanic Blacks with CP-CRE, 101 (64.7%) were nursing home residents, whereas 281 (71.1%) among the 395 non-Hispanic Whites were nursing home residents. Also, 64 Non-Hispanic Blacks (41%) died within 1 year of their first specimen collection date compared to 92 Non-Hispanic Whites (23.3%). Non-Hispanic Blacks with CP-CRE who died within 1 year had a mortality rate of 5.6 per 100,000 (95% CI, 4.21–6.94) Black population, which was 1.6 times higher than Non-Hispanic White persons at 3.5 per 100,000 (95% CI, 2.94–3.95; χ^2^ *P* < .001) White population. **Conclusions:** Despite a lower mean age, non-Hispanic Black CP-CRE cases had a higher 1-year mortality rate than non-Hispanic Whites. Racial and ethnicity data often are missing or incomplete from surveillance data. Data linkages can be a valuable tool to gather additional clinical and demographic data that may be missing from public health surveillance data to improve our understanding of health disparities. Recognition of these health disparities among CRE can provide an opportunity for public health to create more targeted interventions and educational outreach.

**Funding:** None

**Disclosures:** None

